# Approval of Coercion in Psychiatry in Public Perception and the Role of Stigmatization

**DOI:** 10.3389/fpsyt.2021.819573

**Published:** 2022-01-07

**Authors:** Sahar Steiger, Julian Moeller, Julia F. Sowislo, Roselind Lieb, Undine E. Lang, Christian G. Huber

**Affiliations:** ^1^University Psychiatric Clinics Basel, University of Basel, Basel, Switzerland; ^2^Division of Clinical Psychology and Epidemiology, Department of Psychology, University of Basel, Basel, Switzerland

**Keywords:** mental illness stigma, social distance, perceived dangerousness, coercive measures, population survey

## Abstract

**Background:** Coercion is routinely used in psychiatry. Its benefits and drawbacks are controversially debated. In addition, the majority of persons with mental health problems are exposed to stigmatization and are assumed to be dangerous. Stigmatization is associated with negative consequences for individuals with mental illness such as disapproval, social rejection, exclusion, and discrimination. Being subjected to coercive measures can increase the stigmatization of the affected persons, and stigmatization might lead to higher approval for coercion.

**Aims of the Study:** This study aims to examine the approval for coercive measures in psychiatry by the general public, and to explore its relation with person- and situation-specific factors as well as with stigmatization.

**Method:** We conducted a representative survey of the general population (*N* = 2,207) in the canton of Basel-Stadt, Switzerland. Participants were asked to read a vignette depicting psychopathological symptoms of a fictitious character and indicate whether they would accept coercive measures for the person in the vignette. Desire for social distance and perceived dangerousness were assessed as measures of stigmatization.

**Findings:** The person in the case vignette exhibiting dangerous behavior, showing symptoms of a psychotic disorder, being perceived as dangerous, and treatment being understood as helpful increased approval of coercion in general, while familiarity of the respondents with mental illness decreased approval.

**Conclusions:** The public attitude regarding the approval of coercion in psychiatry is highly differentiated and largely follows the current legal framework and medical treatment guidelines. Higher approval occurred in situations of self-harm or harm to others and when coercive measures were thought to have a beneficial effect for the affected persons. A considerable part of the approval for coercion is predicted by stigmatization. With the increasing severity of coercive measures, the influence of person- and situation-specific factors and of familiarity with mental illness decreased and generalizing and stigmatizing attitudes became stronger predictors for the approval of more severe measures.

## Introduction

Coercive measures are still common in psychiatric emergencies. Although they restrict patients' autonomy in medical decision making and can have detrimental effects on the affected persons and their health-related outcomes, the use of such measures can be legally and clinically justified in situations where no others measures are available to avoid harm to the patient or others (1–3). The frequency of coercive measures varies markedly, both internationally and locally, due to differences in legislation, regulations, and clinical practice among and within countries (4, 5). However, patient-centered and supportive treatment settings and an implementation of open-door strategies seem successful in a responsible reduction of coercive measures (6, 7).

Numerous studies have shown inconsistency in patient-related factors that may contribute to an increased risk for coercion. These include diagnosis, level of aggression, sociodemographic, and socioeconomic characteristics. Concerning diagnosis, previous studies revealed that people with psychotic disorders are at high risk for coercive measures (8–10). Others found that patients with personality disorders (9) or substance use related disorders (11) are the most targeted groups for involuntary psychiatric treatment. The sociodemographic factors are also inconsistent in the literature. Male gender and migratory background are associated with coercion (12–14). However, regarding gender, some studies also reported an increased risk for coercive measures in females compared to male patients (15, 16). Concerning dangerousness, numerous studies found that persons with actual or impending danger to self or others are at high risk of compulsory admission (17, 18). This is the case despite the lack of robust empirical evidence for a positive immediate or long-term effect of coercive measures regarding aggression and suicidality. A review of 36 studies, e.g., found insufficient evidence for the short-term effectiveness of coercive measures to reduce danger to others and oneself in psychiatric settings (19).

In addition to research on the risk factors for coercion, some studies evaluated the adverse effects of coercion on patients. Coercive measures were found to be accompanied by negative emotions such as fear, anger, shame, and helplessness for the patients (20, 21) and can be traumatized (22). Moreover, involuntary treatment negatively influences patient-therapist relationships (23) and can lead to poor adherence to therapy (24).

Furthermore, studies found that coercion can induce stigma (25). Stigmatization is associated with negative consequences for individuals with mental illness such as disapproval, social rejection, exclusion, and discrimination (26–28). Furthermore, perceived public stigma constitutes a major barrier to seeking professional help for mental problems, thereby contributing to treatment avoidance (29, 30). Stigma is often described as a “second disease” because the fear of rejection is perceived just as stressful as the condition itself (31). Under the influence of labeling theory, psychiatric stigma research has investigated the negative attitudes of society toward mentally ill people.

Link et al. (32) measured stigma associated with mental illness in terms of social rejection. They demonstrated that perceptions of dangerousness of mentally ill persons affected the way in which participants responded to them and led to more social rejection. Perceived dangerousness thereby provokes a desire to maintain social distance from mentally ill people (33). In the context of the “modified labeling theory,” Link and Castille (34) showed the relation between stigma and coercion. They found that coercion increases stigma, erodes quality of life, and through stigma leads to lower self-esteem. Besides social rejection and perceived dangerousness, researchers have also explored other factors that influence stigmatization. For instance, Angermeyer and Matschinger (35) illustrate that respondents who have contact with individuals with mental illness were tolerant toward mental illness and less likely to believe that the latter are dangerous and unpredictable.

In addition, research on stigma associated with psychiatric symptoms found differences in the levels of stigmatization between different psychiatric diagnoses (36). For instance, individuals with schizophrenia are faced with higher stigmatization and discrimination than people with depression (37–39). However, only a few studies have focused on the stigmatization of individuals with personality disorders. Markham (40) evaluated the effects of the labeled borderline personality disorder (BPD) on staff attitudes and perceptions in the UK. He revealed that social rejection and perceived dangerousness (as indicators for stigma) were higher for BPD than for schizophrenia and depression. Yet, the beliefs of mental health professionals about the dangerousness of BPD might not reflect the attitude of the general population. Thus, more research on how the general population actually stigmatizes patients with BPD compared to other diagnoses is needed.

As stigmatization of persons with mental illness is associated with the attribution of dangerousness (41, 42) and coercion is a measure of last resort in psychiatry used to manage dangerousness (43), it is plausible that higher stigmatization may be associated with higher acceptance of compulsory measures in the population. Approval of coercion might be a negative consequence to people with mental illnesses as a result of labeling dangerousness to them. However, the association of stigmatization of persons with mental illness and the approval of coercion is currently underresearched.

### Aims of the Study

The present study aims to measure the extent to which the general population approves the coercion of individuals with mental disorders. It examines whether established indicators of mental illness stigma such as desire for social distance and perceived dangerousness are associated with the approval of coercion. In addition, we investigated whether the approval of coercion varies regarding the type of mental disorder, familiarity with mental illness, the type of dangerous behavior, or the gender of the mentally ill person. Finally, the public's beliefs about the benefits of coercion were examined regarding their association with the approval of coercive measures.

## Methods

### Samples and Procedures

Data for the current study stem from a vignette-based representative population survey on psychiatric service use and stigmatization that was conducted from autumn 2013 to spring 2014 among citizens of Basel, Switzerland. A sample of 10,000 individuals was randomly drawn from the cantonal resident register and was mailed study material. To be eligible, participants had to have been registered in a private household in the municipality of Basel, Bettingen, or Riehen for a minimum of 2 years, had to be aged between 18 and 65 years, and had to have sufficient knowledge of the German language. This study was approved by the local ethics committee (Ethikkommission Nordwest- und Zentralschweiz, EKNZ 2014-394) and conducted according to the Declaration of Helsinki. Informed consent was obtained from all study participants. They agreed to return the completed survey material. Participants were informed about the scope of the study and their rights in an accompanying letter. An email address and hotline telephone number were provided in case the participants needed additional information.

The final sample consisted of 2,207 individuals (61.5% female, 66.5% Swiss citizens), reflecting a response rate of 22.1%. The mean age of the participants was 43.4 years (SD = 13.4). A total of 6.2% percent had completed only 9 years of schooling obligatory in Switzerland, 51.3% had completed secondary education (~12 years), and 42.0% had a university degree. Data from 1,095 participants who had received the clinic vignette could not be entered in the current analyses, as rating approval of coercion was differently operationalized in their questionnaire. This resulted in a final sample size of 1,112 participants.

To assess the representativeness of our sample, respondent characteristics were compared to official census data as published in the statistical Almanac of Basel-City (44). However, this comparison has to be interpreted with caution, as the data available from the statistical almanac represent the whole population of Basel-City without the restrictions posed by our in- and exclusion criteria. At the end of 2013, 191,606 persons were registered in Basel-City. 52.0% were of female gender, 67.0% were Swiss citizens, and 45.7% were single. Mean age was 42.9 years. 17.5% had completed obligatory school, 48.6 secondary education, and 32.5% higher/university education. The comparison shows that questionnaires were sent out to over 5.2% of the population. The study sample represents more than 1.2% of the total population and can be assumed to be representative regarding age, nationality, marital status, and living situation. However, there seems to be an overrepresentation of women and of persons with higher education in our sample.

### Legal Framework for Coercive Measures

According to the cantonal legislation in Basel-Stadt and national legislation in Switzerland, involuntary hospitalization is possible if the following conditions are fulfilled (45): (1) the person is in a state of weakness because of a mental illness or severe neglect, (2) there is a situation of immediate or directly impending danger to the person or others, or the person's actions cause intolerable burden on their environment, (3) hospitalization is the single adequate measure to solve this situation and other less restrictive measures are not available. Involuntary medication is legally allowed for persons with involuntary hospitalization, if (1) without treatment there is an immediate or directly impending risk for the person's health or for the physical integrity and the life of others, (2) the person is not able to correctly assess the need for treatment, (3) and there are no other less restrictive measures available. Seclusion is allowed as a safety measure if (1) it is the only measure available to protect the person himself or others, (2) enable involuntary treatment, (3) or to counter a severe disturbance of social co-existence and there are no other less restrictive measures available. Other coercive measures like restraining patients are not used in the general psychiatric hospital that provides obligatory care for the population of Basel-Stadt (UPK Basel) and where most coercive measures in the canton are performed, and have therefore not been explored in the current study.

Thus, although the questions in the current survey were not directly modeled exactly after the complex legal prerequisites for compulsory measures, they were designed to cover their overarching aspects: a state of weakness due to a mental illness was present in all case vignettes, the presence of danger to persons or others was examined by providing cases with no dangerous behavior, self-endangering behavior or behavior endangering others, and the participants were asked if they deem the compulsory measure useful for treatment.

### Study Material

Study material consisted of written vignettes and questionnaires. Apart from the sociodemographic variables, the questionnaires measured desire for social distance and perceived dangerousness as indicators for stigmatization, familiarity with mental illness, approval of coercion, personality traits and other variables. Vignettes presented a fictitious character and depicted either a psychiatric disorder of the character (case vignette) or a clinic where the character had been admitted to (clinic vignette. Within both types of vignettes, the gender and dangerousness of the fictitious patient were systematically varied. It was explicitly stated that within the last month the main character (case vignette) or the patients at the clinic (clinic vignette) displayed no dangerous behavior, self-endangering behavior, or behavior endangering others.

Additionally, between the case vignettes, the type of psychiatric disorder was systematically varied, which either described a case of acute psychotic disorder, a case of alcohol dependency, or a case of borderline personality disorder. None of these were labeled directly, but they had symptoms fulfilling the DSM-V criteria (46) for the respective disorder. Apart from these characteristics that were systematically varied, all other information was kept constant between the vignettes to eliminate potential confounders. Prior to the main survey, vignettes were submitted to psychiatrists and clinical psychologists (*N* = 18) for blind diagnostic allocation. Supporting the validity of the case vignettes, each diagnosis was labeled correctly by all clinical experts.

Moreover, between the clinic vignettes, the type of psychiatric service institution to which the fictitious character was admitted was also systematically varied. Vignettes either described a general hospital that included a psychiatric unit, or a psychiatric hospital, or a psychiatric hospital that included a forensic unit. There were no significant differences in the number of respondents per individual vignette condition, neither between the different types of case vignettes [*χ*2 (17, *N* = 1,112) = 19.00, *p* = 0.329] nor between the different types of clinic vignettes [*χ*2 (17, *N* = 1,095) = 6.84, *p* = 0.986].

### Measures

The approval of coercive measures was assessed with three items asking whether the participant would accept one of the following coercive measures for the fictitious character in the vignette: (1) involuntary hospitalization, (2) involuntary medication, and (3) seclusion. Responses were made on a 4-point Likert scale (agree strongly, agree a little, disagree strongly, disagree a little). The reliability (Cronbach's alpha) of the three items was 0.86. Approval of any type of coercion was operationalized if the respondent accepted one of the three measures.

Desire for social distance was measured using a modification (32) of the Bogardus Social Distance Scale (47). The scale consists of seven items asking to what degree the respondent would accept each of the following social relationships with the stigmatized person: sublessee, co-worker, neighbor, caretaker of one's child, spouse of a family member, and member of the same social circle. Responses were made on a 4-point Likert scale, with lower values indicating greater acceptance of the person in the vignette (i.e., a lower desire for social distance).

Perceived dangerousness was measured with the dangerousness scale (48, 49). The scale consists of eight items that assess individual beliefs about the dangerousness of the fictitious person in the vignette. Responses were made on a 4-point Likert scale and a composite (with higher values indicating higher perceived dangerousness) was derived by totaling the sum of all items.

Familiarity with mental illness was measured with four items, similar to the approach of Angermeyer and Matschinger (35), respectively, asking whether psychiatric treatment had been undergone by (1) the participant, (2) a family member of the participant, or (3) a friend of the participant, or whether (4) none of these applied. If the criteria for multiple categories were fulfilled, we chose the one indicating the highest familiarity.

### Statistical Analysis

All statistical analyses were conducted using the SPSS 24 statistical package for Windows (IBM Corporation, Armonk, NY, USA). Approval of any type of coercion was defined as the main outcome for the current analyses. Approval of involuntary hospitalization, involuntary medication, and seclusion were chosen as secondary outcomes. We therefore conducted logistic regression analyses with any type of compulsory measure, or with involuntary hospitalization, involuntary medication, and seclusion as dependent binary variables. In the regression analyses, the type of mental disorder, endangering behavior of the fictitious person in the vignette, perceived dangerousness, desire for social distance, respondent's, familiarity with psychiatric illness, gender of the fictitious person, the respondent's gender and whether the respondents believe that treatment would be useful were entered as independent variables. Categorical predictors with more than two categories (i.e., type of mental disorders, degree of familiarity with psychiatric illness, and endangering behavior) were entered as dummy variables. Moreover, we calculated the mean and standard deviation for these three variables (see Table 1). Yet, logistic regression analysis offers a significance test for the difference between the chosen reference category and each of the chosen comparison groups (e.g., psychosis vs. BPD; psychosis vs. alcohol dependency). To compare the dummy variables (e.g., alcohol dependency vs. BPD), we conducted *post hoc* tests with Bonferroni correction to prevent type I error inflation. The level of significance was set at *p* ≤ 0.05.

**Table 1:** Mean (*M*) and standard deviation (*SD*) for endangering behavior, diagnosis and familiarity in dependence of coercive measures.

	**Any type of coercion**		**Involuntary hospitalization**		**Involuntary medication**		**Isolation**	
	***M***	***SD***	***M***	***SD***	***M***	***SD***	***M***	***SD***
**Endangering behavior**								
None	0.162	0.369	0.139	0.346	0.097	0.296	0.025	0.156
Self-endangering	0.293	0.456	0.228	0.420	0.180	0.385	0.054	0.227
Endangering others	0.289	0.454	0.216	0.412	0.175	0.381	0.082	0.275
**Diagnosis**								
Psychosis	0.315	0.465	0.262	0.440	0.206	0.405	0.054	0.227
Alcohol dependency	0.207	0.406	0.159	0.366	0.112	0.316	0.048	0.213
BPD	0.220	0.415	0.162	0.369	0.133	0.340	0.058	0.234
**Familiarity**								
Friend	0.214	0.411	0.171	0.377	0.136	0.343	0.050	0.219
Family	0.241	0.428	0.190	0.393	0.143	0.351	0.049	0.216
Self	0.240	0.428	0.180	0.385	0.151	0.359	0.045	0.207

## Results

### Approval of Any Type of Coercive Measure

The total model containing all predictors was significant (*N* = 1,112, *χ*^2^ = 149.3, *df* = 11, *p* < 0.001). It explained 19.6% of the variance in approval of coercive measures (Nagelkerke *R*^2^ = 0.196). The logistic regression analysis revealed that endangering behavior, perceived dangerousness, type of psychiatric diagnosis, familiarity with mental illness, and whether treatment was perceived as useful were significant predictors (see Table 2).

**Table 2:** Logistic regression model for approval of any type of coercion.

					***CI***	
	***B***	***SE***	***p***	***OR***	**Lower**	**Upper**
**Endangering behavior**						
None vs. self-endangering	0.748	0.198	<0.001	2.112	1.433	3.115
None vs. endangering others	0.477	0.207	0.021	1.611	1.074	2.418
**Diagnosis**						
Psychosis vs. alcohol dependency	−0.726	0.193	<0.001	0.484	0.332	0.706
Psychosis vs. BPD	−0.516	0.187	0.006	0.597	0.413	0.862
**Familiarity**						
Friends vs. none	−0.730	0.269	0.007	0.482	0.285	0.816
Family vs. none	−0.467	0.256	0.068	0.627	0.379	1.035
Self vs. none	−0.386	0.256	0.132	0.680	0.411	1.123
Desire for social distance	0.038	0.026	0.144	1.038	0.987	1.092
Perceived dangerousness	0.137	0.024	<0.001	1.146	1.094	1.201
Treatment useful	1.543	0.330	<0.001	4.680	2.453	8.928
Gender (vignette)	0.169	0.156	0.279	1.184	0.872	1.609
Gender (respondent)	0.189	0.153	0.216	1.208	0.895	1.631
Constant	−4.649	0.614	0.000	0.010		

*B = unstandardized regression weight, SE, standard error; CI, Confidence interval; p, p-value; OR, odds ratio; BPD, borderline personality disorder; vs., versus. R^2^ = 0.196 (p < 0.001)*.

Regarding endangering behavior, information that the fictitious person endangers her-/himself (*B* = 0.748, *p* < 0.001) or others (*B* = 0.477, *p* = 0.021) was significantly associated with more approval of coercion than information that the fictitious person endangers no one at all. Furthermore, a Bonferroni-adjusted *post hoc* analysis revealed a significant mean difference (*MD*) in the approval of coercive measures between information that the fictitious person endangers her- /himself (*MD* = 0.131, *p* < 0.001; 95%-CI [0.099, 0.163]) or others (*MD* = 0.128, *p* < 0.001; 95%-CI [0.095, 0.160]) vs. information that the fictitious person endangers no one at all. However, there was no significant mean difference between the information that the fictitious person endangers her- /himself vs. information that the fictitious person endangers others (*MD* = 0.004, *p* > 0.05, 95%-CI [−0.028, 0.037]).

Concerning the type of psychopathological symptoms, a Bonferroni-adjusted *post hoc* analysis showed that an acute psychotic disorder was significantly associated with more approval of coercion than symptoms of BPD (*MD* = 0.095, *p* = 0.009; 95%-CI [0.063, 0.127]) and those of alcohol dependency (*MD* = 0.108, *p* = 0.002; 95%-CI [0.076, 0.139). However, the approval of coercive measures did not significantly differ between BPD and alcohol dependency (*MD* = 013, *p* > 0.05, 95%-CI [−0.019, 0.045]).

Concerning familiarity, a friend of the respondent having undergone psychiatric treatment was significantly associated with less acceptance of coercive measures compared to no familiarity with psychiatric treatment (*B* = −0.730, *p* = 0.007). A Bonferroni-adjusted *post hoc* analysis showed no significant mean difference (*MD*) in approval of coercive measures between participant her-/himself vs. a family member (*MD* = 0.0005, *p* > 0.05, 95%-CI [−0.046, 0.045]), participant her- /himself vs. a friend (*MD* = 0.026, *p* > 0.05, 95%-CI [−0.024, 0.076]) or a family member vs. a friend having undergone psychiatric treatment (*MD* = 0.027, *p* > 0.05, 95%-CI [−0.023, 0.077]).

Perceived dangerousness (*B* = 0.137, *p* < 0.001) and whether treatment was perceived as useful (*B* = 1.543, *p* < 0.001) were positively associated with approval of coercion. The results indicate that when participants perceived the fictitious person as dangerous and when they deem that coercion is useful to the mentally ill person, they were more likely to approve coercive measures. Finally, gender of the fictitious person, the participant's gender, and desire for social distance were not significantly associated with the approval of coercive measures in general.

### Approval of Involuntary Hospitalization and Involuntary Medication

Tables 3, 4 show the secondary analyses for involuntary hospitalization and involuntary medication. The model for involuntary hospitalization was significant (*N* = 1,112, *χ*^2^ = 130.4, *df* = 11, *p* < 0.001). It explained 18% of the variance in approval of coercive measures (Nagelkerke *R*^2^ = 0.182). Respectively, the model for involuntary medication was significant (*N* = 1,112, *χ*^2^ = 96.8, *df* = 11, *p* < 0.001). It explained 15% of the variance in approval of coercive measures (Nagelkerke *R*^2^ = 0.151).

**Table 3:** Logistic regression model for approval of involuntary hospitalization.

					***CI***	
	***B***	***SE***	***p***	***OR***	**Lower**	**Upper**
**Endangering behavior**						
None vs. self-endangering	0.554	0.210	0.008	1.740	1.153	2.627
None vs. endangering others	0.169	0.222	0.445	1.185	0.767	1.829
**Diagnosis**						
Psychosis vs. alcohol dependency	−0.780	0.208	<0.001	0.458	0.305	0.689
Psychosis vs. BPD	−0.583	0.202	0.004	0.558	0.376	0.829
**Familiarity**						
Friends vs. none	−0.784	0.281	0.005	0.457	0.263	0.793
Family vs. none	−0.546	0.266	0.040	0.579	0.344	0.976
Self vs. none	−0.601	0.269	0.026	0.548	0.324	0.929
Desire for social distance	0.043	0.028	0.122	1.044	0.989	1.103
Perceived dangerousness	0.130	0.026	<0.001	1.139	1.083	1.197
Treatment useful	1.840	0.412	<0.001	6.299	2.809	14.126
Gender (vignette)	0.160	0.168	0.343	1.173	0.843	1.632
Gender (respondent)	0.201	0.164	0.220	1.223	0.886	1.688
Constant	−4.942	0.686	0.000	0.007		

*B = unstandardized regression weight, SE, standard error; CI, Confidence interval; p, p-value; OR, odds ratio; BPD, borderline personality disorder; vs., versus. R^2^ = 0.182 (p < 0.001)*.

**Table 4:** Logistic regression model for approval of involuntary medication.

					***CI***	
	***B***	***SE***	***p***	***OR***	**Lower**	**Upper**
**Endangering behavior**						
None vs. self-endangering	0.696	0.240	0.004	2.007	1.253	3.213
None vs. endangering others	0.417	0.249	0.095	1.517	0.930	2.472
**Diagnosis**						
Psychosis vs. alcohol dependency	−0.996	0.233	<0.001	0.369	0.234	0.583
Psychosis vs. BPD	−0.621	0.218	0.004	0.537	0.350	0.824
**Familiarity**						
Friends vs. none	−0.301	0.313	0.337	0.740	0.401	1.367
Family vs. none	−0.165	0.301	0.584	0.848	0.470	1.529
Self vs. none	0.006	0.299	0.984	1.006	0.560	1.808
Desire for social distance	0.086	0.031	0.006	1.090	1.026	1.158
Perceived dangerousness	0.107	0.028	<0.001	1.113	1.054	1.175
Treatment useful	0.938	0.356	0.008	2.555	1.271	5.138
Gender (vignette)	0.124	0.185	0.501	1.132	0.788	1.626
Gender (respondent)	0.043	0.182	0.812	1.044	0.730	1.493
Constant	−4.987	0.713	0.000	0.007		

*B = unstandardized regression weight, SE, standard error; CI, Confidence interval; p, p-value; OR, odds ratio; BPD, borderline personality disorder; vs., versus. R^2^ = 0.151 (p < 0.001)*.

Self-endangering behavior was significantly associated with acceptance of involuntary hospitalization (*B* = 0.554, *p* = 0.008) and involuntary medication (*B* = 0.696, *p* = 0.004). Additionally, a Bonferroni-adjusted *post hoc* test showed no significant mean difference (*MD*) between information that the fictitious person endangers her-/himself vs. information that the fictitious person endangers others regarding the approval of involuntary hospitalization (*MD* = 0.012, *p* > 0.05, 95%-CI [−0.018, 0.041]) or medication (*MD* = 0.005, *p* > 0.05, 95%-CI [−0.022, 0.032]).

Concerning the type of psychiatric disorders, a Bonferroni-adjusted *post hoc* analysis revealed that an acute psychotic disorder was significantly associated with more approval of involuntary hospitalization than symptoms of BPD (*MD* = 0.099, *p* = 0.002; 95%-CI [0.071, 0.129]) and those of alcohol dependency (*MD* = 0.102, *p* = 0.001; 95%-CI [0.073, 0.132). However, the approval of involuntary hospitalization did not significantly differ between BPD and alcohol dependency (*MD* = 0.002, *p* > 0.05, 95%-CI [−0.027, 0.032]).

Similarly, acute psychotic disorder was significantly associated with more approval of involuntary medication than symptoms of BPD (*MD* = 0.074, *p* = 0.015; 95%-CI [0.048, 0.100]) and those of alcohol dependency (*MD* = 0.094, *p* = 0.001; 95%-CI [0.068, 0.121). However, the approval of involuntary medication did not significantly differ between BPD and alcohol dependency (*MD* = 0.021, *p* > 0.05, 95%-CI [−0.006, 0.047]).

Perceived dangerousness was significantly associated with more acceptance of involuntary hospitalization (*B* = 0.130, *p* < 0.001) and medication (*B* = 0.107, *p* < 0.001). In addition, when participants perceived coercion as useful for the person in the vignette, they were more likely to accept involuntary hospitalization (*B* = 1.84, *p* < 0.001) and medication (*B* = 0.938, *p* = 0.008).

Familiarity with mental illness showed inhomogeneous results and was significantly associated with approval of involuntary hospitalization, but not of involuntary medication. A Bonferroni-adjusted *post hoc* analysis showed no significant mean difference (*MD*) in approval of involuntary hospitalization between participant her- /himself vs. a family member (*MD* = −0.010; *p* > 0.05, 95%-CI [−0.052, 0.032]), participant her- /himself vs. a friend (*MD* = 0.009, *p* > 0.05, 95%-CI [−0.037, 0.054) or a family member vs. a friend (*MD* = 0.019, *p* > 0.05, 95%-CI [−0.027, 0.065]) having undergone psychiatric treatment.

Inhomogeneous results were also found for a desire for social distance, which was significantly associated with the approval of involuntary medication (*B* = 0.086, *p* = 0.006), but not with the approval of involuntary hospitalization. The gender of the fictitious person and the participant's gender were, again, not significantly associated with the approval of involuntary hospitalization and involuntary medication.

### Approval of Seclusion

Regression analysis for the secondary outcome seclusion is presented in Table 5. The total model containing all predictors was significant (*N* = 1,112, *χ*^2^ = 64.6, *df* = 11, *p* < 0.001). It explained 17% of the variance in approval of coercive measures (Nagelkerke *R*^2^ = 0.174).

**Table 5:** Logistic regression model for approval of seclusion.

					***CI***	
	***B***	***SE***	***p***	***OR***	**Lower**	**Upper**
**Endangering behavior**						
None vs. self-endangering	0.642	0.420	0.126	1.900	0.835	4.324
None vs. endangering others	0.721	0.410	0.079	2.055	0.920	4.590
**Diagnosis**						
Psychosis vs. alcohol dependency	−0.392	0.364	0.282	0.676	0.331	1.380
Psychosis vs. BPD	0.080	0.341	0.815	1.083	0.555	2.113
**Familiarity**						
Friends vs. none	−0.502	0.444	0.259	0.605	0.254	1.446
Family vs. none	−0.291	0.427	0.497	0.748	0.324	1.728
Self vs. none	−0.349	0.432	0.418	0.705	0.303	1.643
Desire for social distance	0.145	0.051	0.004	1.156	1.046	1.278
Perceived dangerousness	0.117	0.043	0.007	1.124	1.033	1.223
Treatment useful	1.723	0.744	0.021	5.599	1.304	24.046
Gender (vignette)	0.131	0.287	0.647	1.140	0.650	2.000
Gender (respondent)	0.271	0.278	0.329	1.311	0.761	2.258
Constant	−8.556	1.259	0.000	0.000		

*B = unstandardized regression weight, SE, standard error; CI, Confidence interval; p, p-value; OR, odds ratio; BPD, borderline personality disorder; vs., versus. R^2^ = 0.174 (p < 0.001)*.

The participants' beliefs about the dangerousness of the fictitious person (*B* = 0.117, *p* = 0.007), their desire to maintain social distance (*B* = 0.145, *p* = 0.004), and if they perceived coercion as useful (*B* = 1.723, *p* = 0.021) were significantly associated with the approval of this compulsory measure. No significant differences emerged for endangering behavior of the person described in the vignette, type of mental disorder, familiarity with the mental illness, and gender of the person in the vignette or the participant.

## Discussion

This vignette-based survey is—to the authors' best knowledge—the first study to examine approval of coercion in the canton of Basel-Stadt, Switzerland, in a representative sample of the general population. Further strengths include the quasi-experimental vignette design, allowing to examine the role of a fictitious person's psychiatric diagnosis, dangerousness to her- /himself or others, and gender for the approval of compulsory measures. Furthermore, the study allowed to investigate the association between approval of coercion and different facets of mental health stigmatization such as desire for social distance and perceived dangerousness.

In accordance with the legal requirements as detailed in the methods section, the opinion that psychiatric treatment is of use for the fictitious person was associated with a higher approval of coercive measures in general and for all individual types of compulsory measures examined. In general, approval of coercive measures in psychiatry by the public seems to be in agreement with the current legal framework and in line with the model of “beneficial coercion” which argues that coercion can be necessary in mental health care to ensure that people with psychiatric disorders who avoid treatment and medication, but are a risk to themselves or others, receive treatment, albeit involuntarily (50).

In addition, when considering any type of coercive measure, self-endangering behavior and behavior endangering others were significantly associated with the public's approval of involuntary measures. However, when specifically examining the different coercive measures explored in the current study, only self-endangering behavior was linked to the approval of involuntary hospitalization and involuntary medication. Yet, endangering behavior was not associated with the approval of seclusion.

Perceived dangerousness, which represents the general attitude that a mentally ill person is unreliable, unpredictable, cannot be trusted, and might be dangerous, was significantly associated with an increased approval of coercion in general and all three individual compulsory measures examined. While both variables are interrelated, endangering behavior, as explicitly outlined in the case vignette pertains to more person- and situation-specific aspects, and perceived dangerousness captures more situation-independent generalizing and stigmatizing attributions.

In our sample, the desire for social distance was not associated with an approval of coercive measures in general and involuntary hospitalization. However, social distance was positively associated with the approval of involuntary medication and seclusion. An interpretation of these findings could be that with higher severity of coercion, person- and situation-specific aspects become less important for the approval of coercive measures by the public, and more generalized attitudes become more prominent predictors of approval. This would be in line with the finding that familiarity with mental illness, a factor known to reduce stigmatization (35, 51), by reframing the assessment of persons and situations based on own experiences, was significantly associated with less approval for coercion in general and for involuntary hospitalization, but did not predict acceptance of involuntary medication or seclusion.

Regarding mental disorders, coercive measures in general, involuntary hospitalization, and involuntary medication were approved more when the fictitious person in the case vignette displayed symptoms of a psychotic disorder than when symptoms of BPD or alcohol dependency were displayed. Pescosolido and Manago (52) found in a recent study that approval of coercion has increased for schizophrenia (but not depression) over the last 22 years. By 2018, over 60 percent of respondents saw people who met criteria for schizophrenia as dangerous to others, and 44–59 percent supported coercive treatment. Interestingly, this estimation of the general population is highly in line with current treatment recommendations, where coercive measures are not recommended in patients with BPD and substance use disorders (53, 54).

Similarly, as seen for endangering behavior, approval of seclusion was not significantly associated with the type of psychiatric diagnosis. Again, a possible explanation for this finding might be that with higher risk for the environment and other persons' integrity, a generalized attitude becomes more prominent by the public.

Finally, although previous research identified high rates of coercive measures for male gender (12), and other studies have shown female persons to be more often exposed to coercion (15), our study revealed no significant difference in approval of coercion regarding the gender of the fictitious person or participants.

### Limitations

A first limitation consists in possible threats to external validity, i.e., the low response rate of 22.1% might account for selection and non-response biases (e.g., reflecting increased participation of women and of persons with higher education). Secondly, the study is based on data from the years 2013/2014. Since then, the public's perception of psychiatry has changed significantly due to intensified media reporting. Moreover, participation was limited to inhabitants of the Swiss canton of Basel-Stadt, which might limit the generalizability of the results. Furthermore, our results are pending replication in other independent surveys. In particular, considering that the regression models were able to explain at most 20% of the variance, there may be several further predictors of approval for coercion in psychiatry that were not examined in the current study. Finally, stigmatization is operationalized in this study by the construct of desired social distance. Actually, it remains unclear to what extent this behavioral intention will translate into concrete behavior.

## Conclusion

In Basel, the public attitude regarding approval of coercion in psychiatry largely follows the current legal framework, with higher approval in situations of self-harm or harm to others and when coercive measures are thought to have a beneficial effect for the affected persons. In this context, a close cooperation with additional inputs of the general population in scientific and treatment-associated questions in psychiatry might be seminal and of additional value for the future. However, a considerable part of the approval for coercion is predicted by stigmatization, which align with the modified labeling theory. Therefore, reducing stigmatization and misconceptions about the dangerousness of persons with mental illness and increasing familiarity with psychiatric patients seem a vital and essential task for clinical psychiatry to further decrease coercive measures in the treatment of persons with mental disorders.

## Data Availability Statement

The original contributions presented in the study are included in the article/supplementary files, further inquiries can be directed to the corresponding author/s.

## Ethics Statement

This study was approved by the local Ethics Committee (EKNZ 2014-394) and conducted according to the Declaration of Helsinki. Informed consent was obtained from all study participants. They agreed to return the completed survey material. Participants were informed about the scope of the study and their rights in an accompanying letter.

## Author Contributions

CH and UL designed the study. JS and CH collected the data. SS, JM, JS, and CH analyzed and interpreted the data. SS and CH wrote the initial draft of the paper. JM, JS, RL, and UL critically revised the manuscript for important intellectual content. SS had full access to all data in the study and takes responsibility for the integrity of the data and the accuracy of the data analysis. All authors have contributed to read and approved the final version of the manuscript.

## Funding

This work was supported by a research grant of the University of Basel (DMS2304) to JS and CH. In addition, CH received intramural funding by the UPK Basel Board of Directors, and from an educational and research grant by Takeda Pharma AG, Pfäffikon, Switzerland. The funding body had no role in the design, collection, analysis, or interpretation of the data; in the writing of the manuscript; or in the decision to submit the manuscript for publication.

## Conflict of Interest

The authors declare that the research was conducted in the absence of any commercial or financial relationships that could be construed as a potential conflict of interest.

## Publisher's Note

All claims expressed in this article are solely those of the authors and do not necessarily represent those of their affiliated organizations, or those of the publisher, the editors and the reviewers. Any product that may be evaluated in this article, or claim that may be made by its manufacturer, is not guaranteed or endorsed by the publisher.
