# Corrigendum: Approval of Coercion in Psychiatry in Public Perception and the Role of Stigmatization

**DOI:** 10.3389/fpsyt.2022.881898

**Published:** 2022-03-22

**Authors:** Sahar Steiger, Julian Moeller, Julia F. Sowislo, Roselind Lieb, Undine E. Lang, Christian G. Huber

**Affiliations:** ^1^University Psychiatric Clinics Basel, University of Basel, Basel, Switzerland; ^2^Division of Clinical Psychology and Epidemiology, Department of Psychology, University of Basel, Basel, Switzerland

**Keywords:** mental illness stigma, social distance, perceived dangerousness, coercive measures, population survey

In the original article, there was an error in the number of participants.

A correction has been made to **Methods**, **Samples and Procedures**, paragraph two.

The original text “Data from 11,095 participants who had received the clinic vignette could not be entered in the current analyses, as rating approval of coercion for the fictitious character was differently operationalized in their questionnaire” has been changed to “Data from 1,095 participants who had received the clinic vignette could not be entered in the current analyses, as rating approval of coercion was differently operationalized in their questionnaire.”

The authors apologize for this error and state that this does not change the scientific conclusions of the article in any way. The original article has been updated.

## Publisher's Note

All claims expressed in this article are solely those of the authors and do not necessarily represent those of their affiliated organizations, or those of the publisher, the editors and the reviewers. Any product that may be evaluated in this article, or claim that may be made by its manufacturer, is not guaranteed or endorsed by the publisher.

